# DNA Methylation in *Ensifer* Species during Free-Living Growth and during Nitrogen-Fixing Symbiosis with *Medicago* spp.

**DOI:** 10.1128/mSystems.01092-21

**Published:** 2022-01-04

**Authors:** George C. diCenzo, Lisa Cangioli, Quentin Nicoud, Janis H. T. Cheng, Matthew J. Blow, Nicole Shapiro, Tanja Woyke, Emanuele G. Biondi, Benoît Alunni, Alessio Mengoni, Peter Mergaert

**Affiliations:** a Department of Biology, Queen’s University, Kingston, Ontario, Canada; b Department of Biology, University of Florence, Florence, Italy; c Université Paris-Saclay, CEA, CNRS, Institute for Integrative Biology of the Cell, Gif-sur-Yvette, France; d U.S. Department of Energy Joint Genome Institute, Berkeley, California, USA; University of Dundee

**Keywords:** rhizobia, symbiotic nitrogen fixation, DNA methylation, cell cycle regulation, CcrM

## Abstract

Methylation of specific DNA sequences is ubiquitous in bacteria and has known roles in immunity and regulation of cellular processes, such as the cell cycle. Here, we explored DNA methylation in bacteria of the genus *Ensifer*, including its potential role in regulating terminal differentiation during nitrogen-fixing symbiosis with legumes. Using single-molecule real-time sequencing, six genome-wide methylated motifs were identified across four *Ensifer* strains, five of which were strain-specific. Only the GANTC motif, recognized by the cell cycle-regulated CcrM methyltransferase, was methylated in all strains. In actively dividing cell cultures, methylation of GANTC motifs increased progressively from the *ori* to *ter* regions in each replicon, in agreement with a cell cycle-dependent regulation of CcrM. In contrast, there was near full genome-wide GANTC methylation in the early stage of symbiotic differentiation. This was followed by a moderate decrease in the overall extent of methylation and a progressive decrease in chromosomal GANTC methylation from the *ori* to *ter* regions in later stages of differentiation. Based on these observations, we suggest that CcrM activity is dysregulated and constitutive during terminal differentiation, which we hypothesize is a driving factor for endoreduplication of terminally differentiated bacteroids.

**IMPORTANCE** Nitrogen fixation by rhizobia in symbiosis with legumes is economically and ecologically important. The symbiosis can involve a complex bacterial transformation—terminal differentiation—that includes major shifts in the transcriptome and cell cycle. Epigenetic regulation is an important regulatory mechanism in diverse bacteria; however, the roles of DNA methylation in rhizobia and symbiotic nitrogen fixation have been poorly investigated. We show that aside from cell cycle regulation, DNA methyltransferases are unlikely to have conserved roles in the biology of bacteria of the genus *Ensifer*. However, we present evidence consistent with an interpretation that the cell cycle methyltransferase CcrM is dysregulated during symbiosis, which we hypothesize may be a key factor driving the cell cycle switch in terminal differentiation required for effective symbioses.

## INTRODUCTION

Methylation of genomic DNA is a pervasive phenomenon found in eukaryotes (1–3), archaea (4), and bacteria (4, 5). The biological roles of DNA methylation are most extensively studied in mammals, where it contributes to normal development and disease via its impact on gene expression (6). In bacteria, DNA methylation is best known for its role in restriction-modification (R-M) systems that are thought to provide defense against phage infection and limit horizontal gene transfer through the degradation of invading nonmethylated DNA (7). Several methyltransferases (MTases) of R-M systems have also been implicated in phase variation in pathogens through modulating gene expression (8). A recent study of over 200 bacterial and archaeal species identified orphan MTases not belonging to R-M systems in nearly half of the genomes (4). To date, biological functions have been attributed to very few orphan MTases, namely, the Dam MTase of the *Gammaproteobacteria* and the CcrM MTase of the *Alphaproteobacteria* (9). The Dam MTase of Escherichia coli is notable for its role in regulation of DNA replication (10, 11) and DNA repair (12) by modulating the activity of other DNA-binding proteins. The CcrM MTase was first identified in Caulobacter crescentus (13), with homologs since identified in diverse *Alphaproteobacteria* (14, 15). CcrM activity was shown to be cell cycle regulated in C. crescentus and Agrobacterium tumefaciens (13, 16), leading to methylation of its cognate DNA motif (the pentanucleotide GANTC) specifically during a short period at the end of DNA replication. This leads to a switching of GANTC sites between fully methylated (methylated on both strands) and hemi-methylated (methylated only on the template strand) as a result of DNA replication (17), which serves to modulate gene expression in a cell cycle-dependent fashion (18–20). Over- and underexpression of *ccrM* result in defects in DNA replication and cell division (14, 16, 19), while its complete loss is lethal under some conditions.

The rhizobia are a polyphyletic group of *Alphaproteobacteria* and *Betaproteobacteria* that can both live free in the soil and enter into an endosymbiotic interaction with legumes (21). This interaction begins following an exchange of signals between the free-living partners (22), and it culminates in the formation of a new organ known as a root nodule, within which the cytoplasm of plant cells contain thousands of N_2_-fixing bacteria called bacteroids. Bacteroid formation results in the differential expression of more than a thousand genes (23, 24) and global changes in cellular metabolism (25). In legumes of the inverted-repeat lacking clade (IRLC) and the dalbergioid clade of the family *Papilionoideae*, bacteroid development involves an additional process of terminal differentiation (26, 27); in other legume clades, bacteroid differentiation is less pronounced and is reversible. Terminal bacteroid development, in contrast to reversible bacteroid formation, involves cell enlargement (bacteroids are 5- to 10-fold longer than their free-living counterparts) and genome endoreduplication (resulting in up to 24 copies of the genome per cell), indicative of a cell cycle transition occurring during differentiation (26). Indeed, the correct expression of cell cycle regulators in *Ensifer* (syn. *Sinorhizobium*) *meliloti*, a symbiont of *Medicago* species of the IRLC, is essential for the formation of functional bacteroids (28, 29), while overexpression of CcrM or disruption of the master cell cycle regulator CtrA can give rise to bacteroid-like morphology in free-living cells (14, 30). Additionally, mutants in the *E. meliloti* cell cycle regulators *divJ*, *cbrA*, and *cpdR1*, encoding three negative regulators of CtrA, form nonfunctional nodules in which bacteroids do not differentiate properly (28, 29, 31), and genes encoding several cell cycle regulators (including CcrM) are strongly downregulated in bacteroids (24). The differentiation and cell cycle switch of bacteroids is controlled by the legume host through the production of a large family of peptides, known as nodule-specific cysteine-rich (NCR) peptides (32–34).

Multiple studies have provided evidence that changes in the methylation status of the DNA of legume nodule cells contribute to symbiotic development (35–37). Conversely, it remains unknown if methylation of rhizobium DNA contributes to the regulation of N_2_-fixation or bacteroid development. We are aware of only one study (38) comparing DNA methylation of a rhizobium (Bradyrhizobium diazoefficiens USDA110) between free-living and symbiotic states (soybean nodules) and comparing these changes with differential expression data. Intriguingly, the authors identified a DNA motif that was methylated specifically in bacteroids (38). However, no clear evidence was presented that methylation of this (or any other) motif is involved in transcriptional regulation, and the number of genes both differentially expressed and differentially methylated in bacteroids did not appear to be different than expected by chance. While these data may suggest that DNA methylation does not play a major role in regulating N_2_-fixation by rhizobia, they do not address the role of DNA methylation in terminal bacteroid differentiation, as *B. diazoefficiens* undergoes reversible differentiation in soybean nodules (39).

Here, we use Pacific Biosciences single-molecule real-time (SMRT) sequencing to detect genome-wide patterns of DNA methylation in four strains belonging to the genus *Ensifer*. Our results indicate that DNA methylation is poorly conserved across the genus, and interestingly, they reveal that the pattern of GANTC methylation in N_2_-fixing bacteroids differs from that of free-living cells. Based on these data, we hypothesize that constitutive activation of the CcrM MTase may be a contributing factor driving terminal differentiation.

## RESULTS

### The methylomes of the genus *Ensifer*.

Our experimental design, as summarized in Materials and Methods and Fig. S1 (at doi.org/10.6084/m9.figshare.16556205), was developed to support an investigation into multiple potential roles of DNA methylation in plant-associated bacteria from the genus *Ensifer* through the use of SMRT sequencing. This was accomplished by (i) including DNA samples isolated from phylogenetically diverse wild-type strains, (ii) examining DNA methylation in a single strain across multiple conditions (exponential-phase growth versus stationary phase; growth with sucrose versus growth with succinate), (iii) investigating the impact of a large-scale genome reduction on DNA methylation patterns, and (iv) isolating DNA from bacteroids purified from legume nodules.

We began with base modification analyses of four wild-type strains from three species, including three nodule-forming strains (*E. meliloti* Rm2011, *E. meliloti* FSM-MA, Ensifer fredii NGR234) and one plant-associated, nonsymbiotic strain (Ensifer adhaerens OV14). To ensure consistency, all strains were grown to mid-exponential phase in a common minimal medium with succinate as the carbon source. A total of six methylated motifs were identified, of which five were m6A modifications and one was a m4C modification (Table 1). Five of the six motifs were methylated specifically in one strain. Only the GANTC motif, recognized by the highly conserved cell cycle-regulated CcrM methyltransferase (13, 14), was methylated in all four strains. To further examine the conservation, or lack thereof, of DNA modification across the genus *Ensifer*, we examined the distribution of methyltransferases in the model species *E. meliloti*. Based on gene annotations, we identified 24 genes encoding putative MTases in a previous pangenome analysis of 20 *E. meliloti* strains (Table S1 at doi.org/10.6084/m9.figshare.16556205) (40). Of these 24 genes, only 1 (*ccrM*) was found in all 20 strains, while 4 were found in 2 strains and 19 were found in a single strain. These results suggest that DNA methylation is unlikely to play a conserved role in the genus *Ensifer* aside from cell cycle control via CcrM-mediated methylation and phage defense.

**TABLE 1:** Methylated motifs identified in this study

Motif^*a*^	Type^*b*^	Count^*c*^	Frequency (motifs/kb)
*E. meliloti* 2011			
G**A**NTC CTN**A**G	m6A	11,169	1.67
RCG**C**CTC YGCGGAG	m4C	3,943	0.59
CGC**A**(N5)GTG GCGT(N5)C**A**C	m6A	1,085	0.16
*E. meliloti* FSM-MA			
G**A**NTC CTN**A**G	m6A	11,215	1.67
TCG**A**(N8)TCGA AGCT(N8)**A**GCT	m6A	2,612	0.39
*E. fredii* NGR234			
G**A**NTC CTN**A**G	m6A	11,111	1.61
CAG**A**(N7)GTTG GTCT(N7)C**A**AC	m6A	188	0.03
*E. adhaerens* OV14			
G**A**NTC CTN**A**G	m6A	8,475	1.10
WNCCG**A**TG WNGGCTAC	m6A	4,596	0.60

a The methylated nucleotides are indicated in boldface font.

b Indicates whether the modification is an N^6^-methyladenoside (m6A) or N^4^-methylcytosine (m4C).

c The total times the motif appears in the genome, regardless of methylation status.

None of the motifs methylated in *E. meliloti* Rm2011 were enriched in the promoter regions of genes previously shown to be differentially expressed when grown with glucose versus succinate (41). Similarly, except for the GANTC motif as discussed below, no global effect of carbon source (sucrose [glycolytic] versus succinate [gluconeogenic]) was observed on the DNA methylation pattern of *E. meliloti* Rm2011 (Fig. S2 at doi.org/10.6084/m9.figshare.16556205). Moreover, no global differences in DNA methylation were detected between *E. meliloti* Rm2011 and RmP3496, an Rm2011 derivative lacking the pSymA and pSymB replicons that together account for 45% of the genome content of *E. meliloti* (42) (Fig. S3 at doi.org/10.6084/m9.figshare.16556205). These results suggest that at least under the tested conditions, most DNA MTases are not likely to have a regulatory function in the genus *Ensifer*, although they could play regulatory roles in other environments.

### Cell cycle regulation by the CcrM methyltransferase.

**FIG 1:**
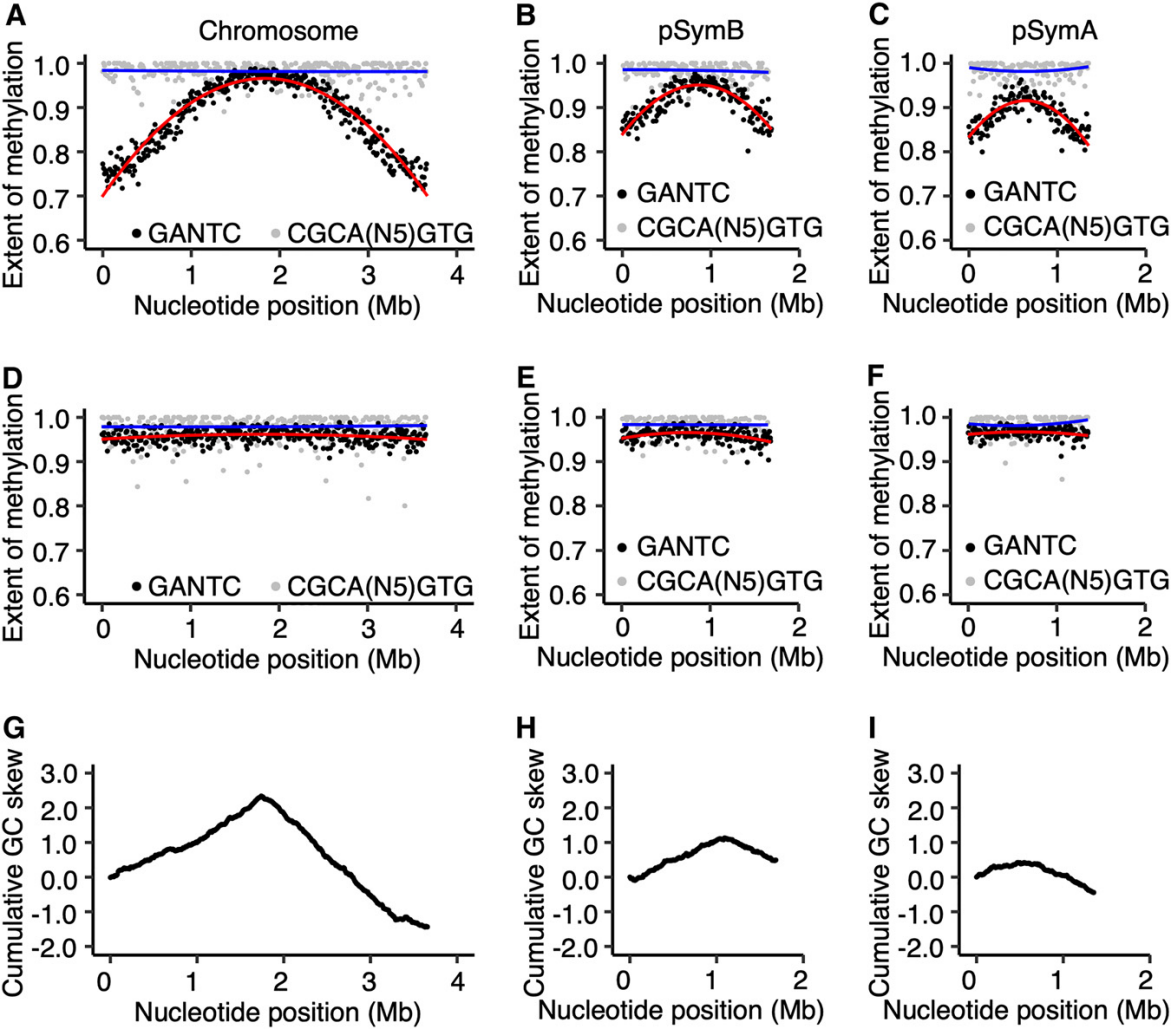
Genome-wide DNA methylation of *E. meliloti* Rm2011. (A to F) The extent of methylation is shown, using a 10-kb sliding window, of GANTC sites (black) and CGCA(N_5_)GTG sites (gray) across the chromosome (A and D), pSymB (B and E), and pSymA (C and F) replicons of exponential phase (A to C) or early stationary phase (D to F) *E. meliloti* Rm2011. Averages from three biological replicates are shown. The red (GANTC) and blue [CGCA(N_5_)GTG] lines are polynomial regression lines calculated in R using the “rlm” method and the formula “y∼poly(x,2).” (G and I) Cumulative GC skews, shown using a 10-kb sliding window, across the *E. meliloti* Rm2011 chromosome (G), pSymB (H), and pSymA (I) replicons.

**FIG 2:**
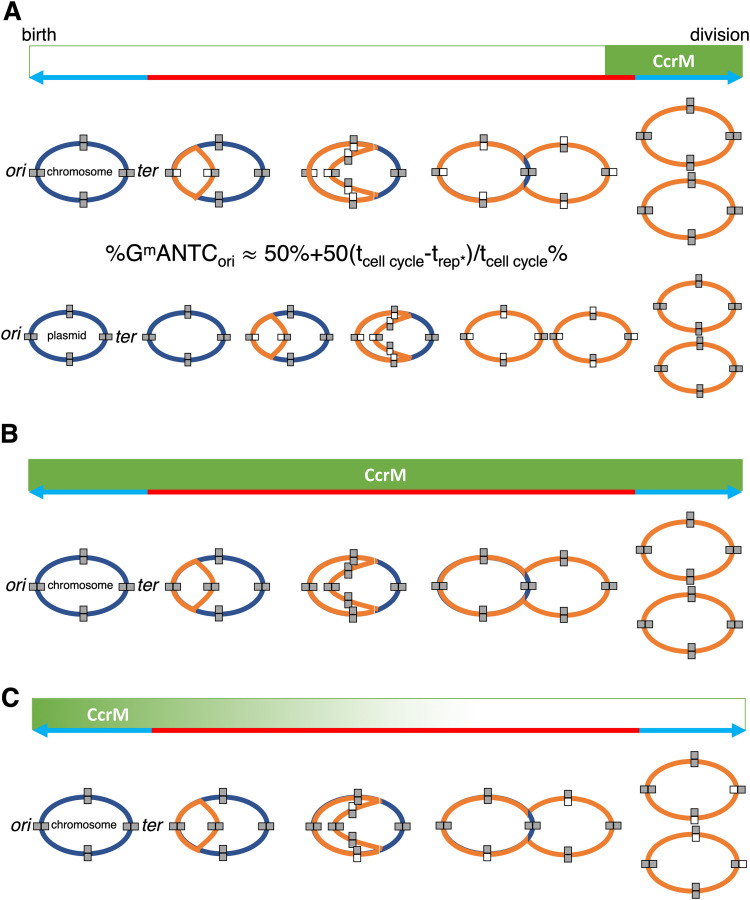
Model describing the GANTC methylation patterns observed in cultures and bacteroids. In all panels, the replication progression and methylation status of the circular chromosome and a megaplasmid are depicted during the cell cycle progression from cell birth until division. The replicons at cell birth are in dark blue, and the newly replicated DNA is in orange. Full-gray rectangles indicate GANTC sites that are in the fully methylated state, while gray/white rectangles indicate the hemimethylated state. The green boxes along the top indicate when CcrM is known or postulated to be active during the cell cycle, while the blue/red line indicates the relevant cell cycle phases; red is the S-phase or genome replication phase and blue is the gap phases and division. (A) The activity of CcrM in free-living cells during exponential growth. The formula expresses the extent of methylation at the origin (*ori*) of replication in an asynchronous bacterial population in culture. (B) The proposed activity of CcrM at an early stage of bacteroid differentiation, during which we suggest that the activity of CcrM is extended to also include the replication phase of the cell cycle. (C) The proposed activity of CcrM at a late stage of bacteroid differentiation, during which we suggest that there is a drop in CcrM activity during the last chromosome replication cycle of the endoreduplication process. The iconography of the illustrations is based on Fig. 1 of Mohapatra et al. (43). Refer to Fig. S22 at doi.org/10.6084/m9.figshare.16556205 for a version of this figure with a more detailed legend.

A progressive increase in the extent of methylation (here defined as the estimated fraction of reads mapping to a motif that were methylated) of GANTC sites was observed from the *ori* to *ter* regions of the chromosomes of all four strains during mid-exponential growth (Fig. 1, and Fig. S4 to S6 at doi.org/10.6084/m9.figshare.16556205). There was a local drop in GANTC methylation around the 1.5-Mb mark in the *E. meliloti* FSM-MA chromosome (Fig. S4 at doi.org/10.6084/m9.figshare.16556205); however, this was seen in only two of three replicates and corresponded to a region of high sequencing depth (Fig. S7 at doi.org/10.6084/m9.figshare.16556205), suggesting the result is a sequencing artifact. In contrast to exponential-phase cultures, GANTC sites displayed near full methylation (averaging ∼95%) across the genome during early stationary phase, while all other motifs displayed near full methylation (averaging 95 to 99%) across the genome regardless of growth state (Fig. 1, and Fig. S4 to S6 at doi.org/10.6084/m9.figshare.16556205). The observed pattern of GANTC methylation indicates a progressive switch from fully to hemi-methylated states as DNA replication proceeds (model provided as Fig. 2), confirming that the CcrM methyltransferase of the family *Rhizobiaceae* is cell cycle regulated as demonstrated in C. crescentus (13, 17, 43). Interestingly, the genome-wide pattern of GANTC methylation displayed a smaller variation in the extent of methylation from the *ori* to *ter* regions in *E. meliloti* Rm2011 when grown with sucrose compared to succinate as the carbon source (Fig. S2 at doi.org/10.6084/m9.figshare.16556205). While this observation could suggest metabolic regulation of CcrM activity, we instead hypothesize, as displayed in Fig. 2A, that it is due to DNA replication being initiated later in the cell cycle when *E. meliloti* is provided sucrose, as recent observations showed that central carbon metabolism influences the rate of DNA polymerase processivity and timing of DNA replication initiation in Bacillus subtilis (44).

Notably, the GANTC methylation pattern differed across replicons within each genome. For example, in *E. meliloti* Rm2011 the extent of GANTC methylation ranged from 0.80 to 0.98 for pSymB and 0.80 to 0.96 for pSymA, while for the chromosome, the range was from 0.71 to 0.99 (Fig. 1). This result suggests that replication of each replicon is asynchronous, with replication of the secondary replicons being initiated later in the cell cycle than that of the chromosome (model provided as Fig. 2).

A previous study identified 462 cell cycle-regulated genes in *E. meliloti* Rm1021 (a near-isogenic relative of strain Rm2011 also derived from the nodule isolate SU47) through RNA sequencing of synchronized cell populations (45), which were classified into six groups based on the timing of their expression. We identified 111 cell cycle-regulated genes, belonging to 78 transcripts, that contained at least 1 GANTC in the predicted promoter regions (defined as the 125 bp upstream of the transcript; Data set S1 at doi.org/10.6084/m9.figshare.16556205), and distribution of these 111 genes across the 6 cell cycle gene expression groups was unbiased (45). As these 111 genes are both cell cycle-regulated and contain a GANTC site, they represent an initial candidate CcrM regulon in *E. meliloti*, although further work is required to identify the CcrM regulon.

Between 31 and 53 GANTC sites were repeatedly not detected as methylated on one or both strands in each of the four wild-type strains (see Data set S2 at doi.org/10.6084/m9.figshare.16556205), as has also been observed in other species (17, 46). However, further work is required to determine if this hypomethylation is biologically meaningful (e.g., CcrM cannot access the site due to binding of another protein [46]) or if it reflects technical artifacts (e.g., low sequencing coverage).

**FIG 3:**
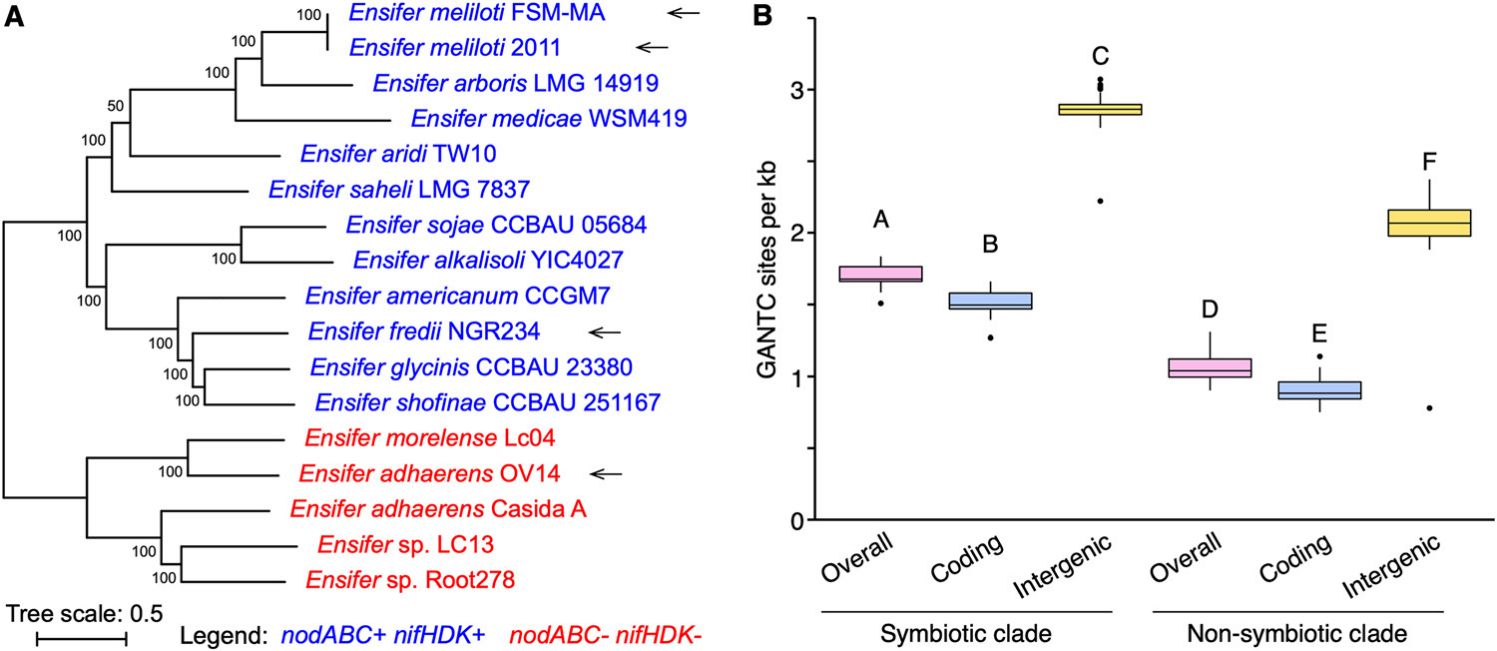
GANTC frequency in the genus *Ensifer*. (A) An unrooted maximum likelihood phylogeny of 17 representative *Ensifer* strains. The phylogeny represents the bootstrap best tree following 100 bootstrap replicates, prepared on the basis of the concatenated nucleotide alignments of 1,566 core genes. Values represent the bootstrap support. N_2_-fixing legume symbionts were identified by the presence of the symbiotic genes *nodABC* and *nifHDK*. They are indicated in blue, while red denotes nonsymbiotic strains. The four wild-type strains used in this study are indicated with arrows. (B) Box plots summarizing the frequency of GANTC sites (presented as GANTC sites per kb) in 157 *Ensifer* strains is shown. The monophyletic “symbiotic” and “non-symbiotic” clades, as defined previously (47), are represented by 113 and 44 genomes, respectively. The densities of GANTC sites across the entire genome (pink), within coding regions (blue), and within intergenic regions (yellow) are shown. Statistically different values (*P < *0.05) are denoted by uppercase letters as determined by a one-way analysis of variance (ANOVA) followed by Tukey’s honestly significant difference (HSD) *post hoc* test.

We found it striking that the *E. adhaerens* OV14 genomes had 2,636 to 2,740 fewer GANTC sites than the other three strains, despite having the largest genome size. Normalized by genome length, there are 1.10 GANTC sites per kb in the *E. adhaerens* OV14 genome (Table 1), which is similar to the 1.12 GANTC sites per kb in C. crescentus NA1000. In contrast, the three legume symbionts contained more than 1.60 GANTC sites per kb across their genomes (Table 1). This result prompted us to examine the frequency and distribution of GANTC sites across 157 *Ensifer* genomes. As defined previously (47), the genus *Ensifer* can be broadly subdivided into two monophyletic clades, the “symbiotic” clade (113 strains), in which nearly all strains are legume symbionts, and the “nonsymbiotic” clade (44 strains), in which nearly all strains are nonsymbionts (Fig. 3A). Consistent with previous results (18), GANTC sites occurred less frequently in all genomes (0.90 to 1.83 GANTC sites per kb) than expected in a random sequence of nucleotides (∼3.5 GANTC sites per kb). Moreover, GANTC sites were ∼2-fold more common in intergenic regions than in coding regions (Fig. 3B). Strikingly, there was a strong and statistically significant difference (*P* value < 1 × 10^−10^; two-sample *t* test) in the frequency of GANTC sites across the genomes of strains belonging to the symbiotic clade compared to strains of the nonsymbiotic clades (Fig. 3B), with an overall average of 1.70 and 1.06 GANTC sites per kb in the symbiotic and nonsymbiotic clades, respectively. The difference in the frequency of GANTC sites between the two clades could not be explained by differences in the GC content of these organisms, as both clades had an average GC content of 61.9% (Fig. S8 at doi.org/10.6084/m9.figshare.16556205), suggesting that the difference reflects underlying differences in the evolution, and possibly the biology, of these two clades.

### The impact of bacteroid differentiation on DNA methylation.

The only previously published study to examine the role of rhizobium DNA methylation during symbiosis using SMRT sequencing did so in a symbiosis where the bacteria do not undergo terminal differentiation (38, 39). To evaluate whether DNA methylation potentially contributes to regulation of terminal differentiation, we determined the DNA methylation patterns of *E. meliloti* Rm2011 and *E. meliloti* FSM-MA bacteroids purified from whole Medicago sativa nodules. *E. meliloti* FSM-MA bacteroids were additionally purified from Medicago truncatula nodules to determine whether the host plant influences bacteroid DNA methylation. *E. meliloti* Rm2011 bacteroids were not isolated from *M. truncatula* nodules as, unlike FSM-MA, Rm2011 forms a poor symbiosis with *M. truncatula* and produces moderately differentiated bacteroids in this host, whereas both strains are equally efficient on *M. sativa* (48, 49). See Kazmierczak et al. for a detailed description of the symbiotic phenotypes of these two strains of *E. meliloti* (49).

Moreover, we exploited the spatially distinct developmental zones that are present in indeterminate nodules (50), like those formed by *M. sativa* and *M. truncatula*. At the tip of these nodules, a bacterium-free meristem is present, responsible for the continuous growth of the nodule. Immediately below is the infection and differentiation zone II, where nodule cells become infected and bacteria differentiate into large, polyploid bacteroids. The tip and zone II of nodules appears white. Adjacent to the white zone II is the easily recognizable pinkish zone III (due to the presence of the oxygen-carrying leghemoglobin), where mature bacteroids fix nitrogen. This nodule tissue organization provided an opportunity to examine how DNA methylation patterns may differ between differentiating and differentiated bacteroids. To this end, *E. meliloti* Rm2011 and FSM-MA bacteroids were isolated from nodules hand-sectioned at the white-pink border; bacteroids isolated from the white sections represent the infecting and differentiating bacteroids (zone II), while those isolated from the pink sections represent the mature, hence terminally differentiated, N_2_-fixing bacteroids (zone III) (see Fig. S9 at doi.org/10.6084/m9.figshare.16556205 for photographs of the plants).

Fluorescence microscopy and flow cytometry confirmed that nodule sectioning resulted in the isolation of distinct bacteroid populations (Fig. S10 to S13 at doi.org/10.6084/m9.figshare.16556205). Nearly all of the bacteroids isolated from whole-nodule samples and zone III samples were enlarged and polyploid, and most were positive for propidium iodide (PI) staining as expected for terminally differentiated bacteroids (26). In contrast, bacteroids of the zone II samples contained a mix of cell types differing in their size, ploidy level, and PI staining. These data confirmed that the whole-nodule samples and zone III samples consisted predominately of mature N_2_-fixing bacteroids, whereas the zone II samples contained a mix of cells at various stages of bacteroid differentiation.

**FIG 4:**
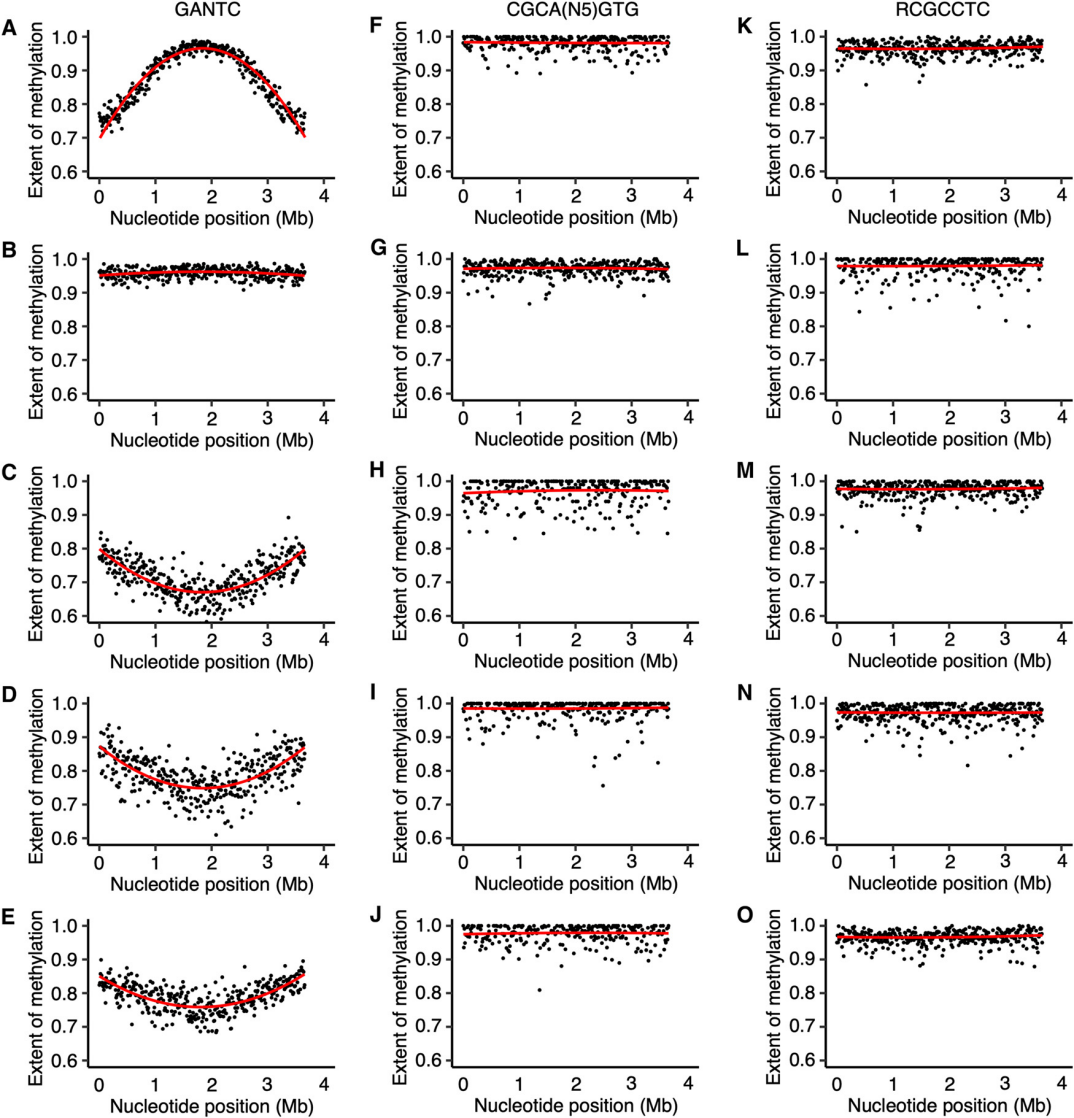
Chromosome-wide DNA methylation of *E. meliloti* Rm2011 bacteroids. (A to O) The extent of methylation of (A to E) GANTC, (F to J) CGCA(N_5_)GTG, and (K to O) RCGCCTC motifs across the *E. meliloti* Rm2011 chromosome is shown using a 10-kb sliding window. Averages from three biological replicates are shown for free-living and whole-nodule samples; the data represent one replicate for the zone II and zone III nodule sections. (A, F, and K) Free-living cells harvested in mid-exponential phase. (B, G, and L) Free-living cells harvested in early stationary phase. (C, H, and M) Bacteroids isolated from *M. sativa* zone II nodule sections. (D, I, and N) Bacteroids isolated from *M. sativa* zone III nodule sections. (E, J, and O) Bacteroids isolated from *M. sativa* whole-nodule samples. The red lines are polynomial regression lines calculated in R using the “rlm” method and the formula “y∼poly(x,2).” Data for pSymB and pSymA are shown in Fig. S14 and S15 at doi.org/10.6084/m9.figshare.16556205.

**FIG 5:**
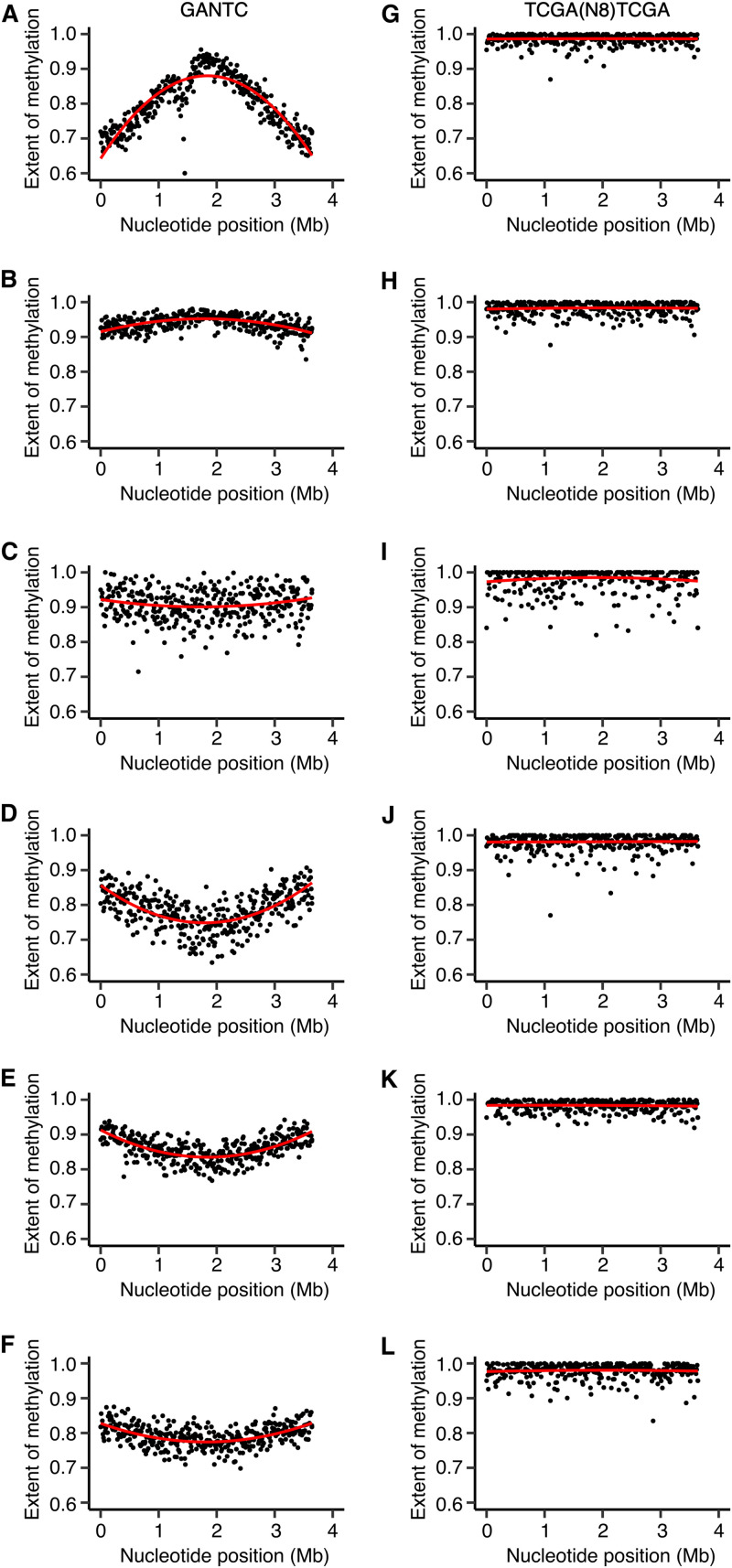
Chromosome-wide DNA methylation of *E. meliloti* FSM-MA bacteroids. (A to L) The extent of methylation of (A to F) GANTC and (G to L) TCGA(N_8_)TCGA motifs across the *E. meliloti* FSM-MA chromosome is shown using a 10-kb sliding window. Averages from three biological replicates are shown for free-living and whole-nodule samples; the data represent one replicate for the zone II and zone III nodule sections. (A and G) Free-living cells harvested in mid-exponential phase. (B and H) Free-living cells harvested in early stationary phase. (C and I) Bacteroids isolated from *M. sativa* zone II nodule sections. (D and J) Bacteroids isolated from *M. sativa* zone III nodule sections. (E and K) Bacteroids isolated from *M. sativa* whole-nodule samples. (F and L) Bacteroids isolated from *M. truncatula* whole-nodule samples. The red lines are polynomial regression lines calculated in R using the “rlm” method and the formula “y∼poly(x,2).” Data for pSymB and pSymA are shown in Fig. S16 and S17 at doi.org/10.6084/m9.figshare.16556205.

With the exception of the GANTC sites (described below), no global difference was observed in the methylation patterns of bacteroids versus free-living cells (Fig. 4 and 5, and Fig. S14 to S17 at doi.org/10.6084/m9.figshare.16556205). Although a lower percentage of each motif was detected as methylated in the bacteroid samples compared to free-living samples, this was correlated with lower sequencing depth (Table S2 at doi.org/10.6084/m9.figshare.16556205) and thus unlikely to be biologically meaningful. Unlike *B. diazoefficiens* USDA110 (38), we did not identify motifs that were methylated specifically in the *E. meliloti* bacteroids. In addition, we found little evidence for any of the methylated motifs being enriched in the promoter regions of *E. meliloti* Rm2011 genes upregulated or downregulated in bacteroids relative to free-living cells, as identified in published transcriptomic data for *E. meliloti* Rm1021 (23). These data suggest that most DNA methylation is unlikely to be a significant factor in directly regulating gene expression in *E. meliloti* bacteroids.

### Bacteroid development influences GANTC methylation.

Bacteroid development involves cell enlargement and genome endoreduplication, indicative of a cell cycle transition occurring during differentiation (26). Indeed, expression of *ccrM* and other genes encoding cell cycle regulators vary across stages of bacteroid development and are strongly downregulated in mature nitrogen-fixing *E. meliloti* bacteroids (24). We were therefore interested in examining whether GANTC methylation by the CcrM MTase was disrupted in bacteroids. Our data revealed a surprising genome-wide pattern of GANTC methylation in *E. meliloti* Rm2011 and FSM-MA bacteroids, which differed from free-living cells in either the exponential or stationary phases of growth (Fig. 4 and 5 and Fig. S14 to S17 at doi.org/10.6084/m9.figshare.16556205). The majority of GANTC sites had moderate to high levels of methylation in zone II, zone III, or whole-nodule samples, averaging 0.71 to 0.95 across each replicon (Fig. 4 and 5 and Fig. S14 to S17 and Tables S3 and S4 at doi.org/10.6084/m9.figshare.16556205). Most distinctively, a progressive decrease in the extent of chromosomal methylation of the GANTC sites was observed from *ori* to *ter* in nearly all bacteroid samples, revealing a characteristic smiling pattern, which differs from the patterns seen in exponential-phase (frowning pattern, i.e., a progressive increase from *ori* to *ter*) and stationary-phase (consistent methylation) cells. The exception was the *E. meliloti* FSM-MA zone II bacteroid sample, which displayed a consistently high level of GANTC methylation across the genome (Fig. 5C). This pattern, which is different from those in exponential-phase cells as well as mature bacteroids, could correspond to the methylation status of an early stage of bacteroid differentiation. We did not observe the same pattern in the *E. meliloti* Rm2011 zone II samples. As noted earlier, the zone II samples contain cells at various stages of differentiation (Fig. S10 to S13 at doi.org/10.6084/m9.figshare.16556205). Given that terminal differentiation is associated with an up to 24-fold increase in DNA content, small increases in the proportion of cells at late stages of differentiation could mask the DNA methylation pattern of the cells at early stages of differentiation. Thus, we hypothesize that the Rm2011 zone II sample captures a later stage of differentiation than that captured by the FSM-MA zone II sample. Supporting this hypothesis, the distribution of DNA content per cell in the flow cytometry data was flatter for *E. meliloti* Rm2011 zone II bacteroids compared to *E. meliloti* FSM-MA zone II bacteroids (Fig. S18 at doi.org/10.6084/m9.figshare.16556205), which suggests that the former sample represents a broader range of differentiation stages than the latter sample. In contrast to GANTC, the extent of methylation of the second m6A modified motif in each genome was consistently high, irrespective of condition or replicon (Fig. 4 and 5 and Fig. S14 to S17 and Tables S3 and S4 at doi.org/10.6084/m9.figshare.16556205). Similarly, sequencing depth was consistent across the length of each replicon (Fig. S7 at doi.org/10.6084/m9.figshare.16556205). These observations indicate that the changes in GANTC methylation patterns are biologically meaningful and not simply a sequencing artifact.

**FIG 6:**
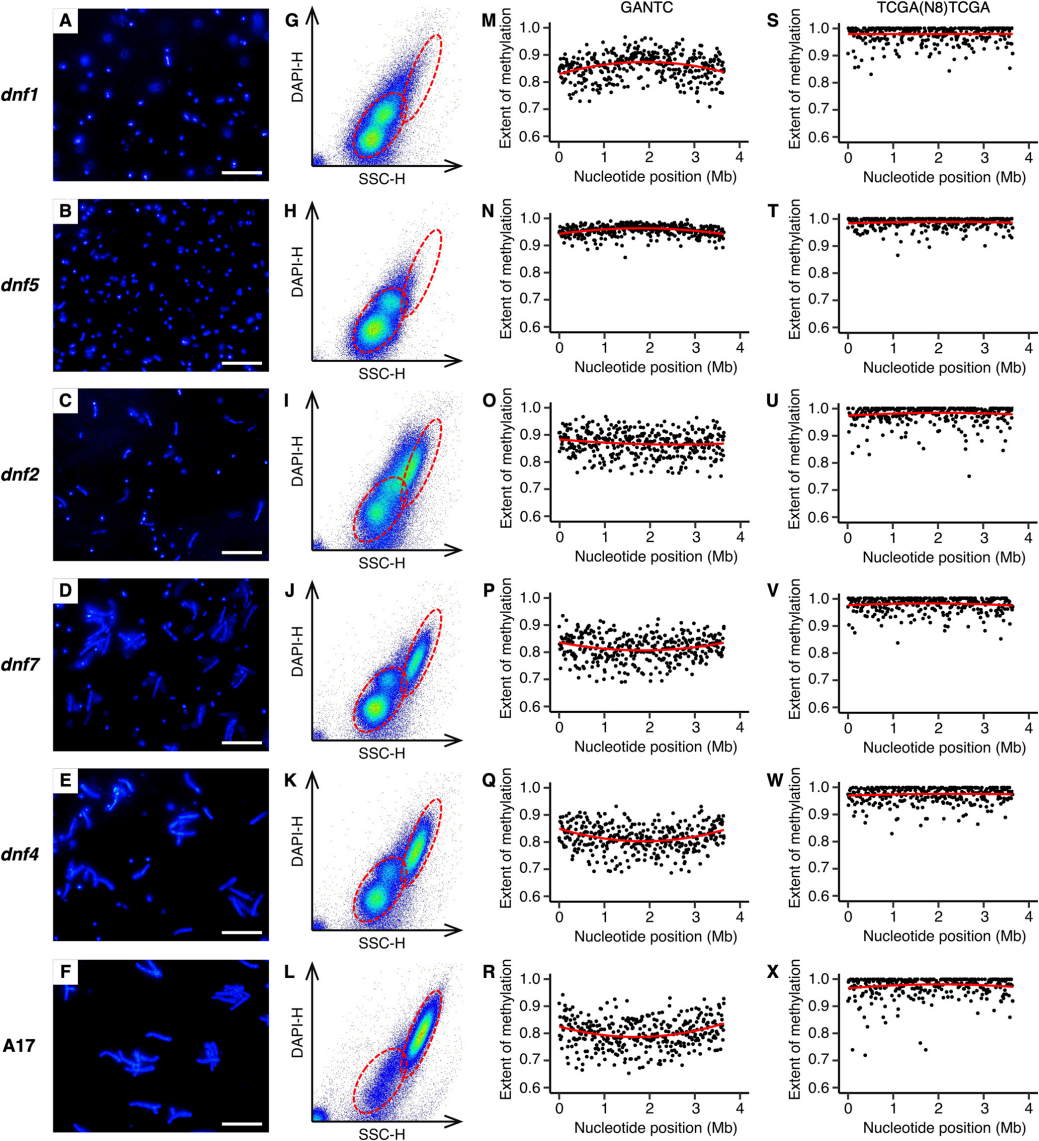
Bacteroid morphology and chromosomal GANTC methylation in *E. meliloti* bacteroids purified from *M. truncatula dnf* mutant nodules. (A to X) Data are shown for *E. meliloti* FSM-MA bacteroids purified from (A, G, M, and S) *M. truncatula dnf1* mutant nodules, (B, H, N, and T) *M. truncatula dnf5* mutant nodules, (C, I, O, and U) *M. truncatula dnf2* mutant nodules, (D, J, P, V) *M. truncatula dnf7* mutant nodules, (E, K, Q, and W) *M. truncatula dnf4* mutant nodules, and (F, L, R, and X) *M. truncatula* A17 wild-type nodules. (A to F) Micrographs of *E. meliloti* FSM-MA bacteroids stained with the DNA binding dye DAPI. The scale bar represents 30 μm. (G to L) Pseudocolored scatterplots displaying the cell morphology (*x* axis) and DNA content (*y* axis) of *E. meliloti* FSM-MA bacteroids, as determined based on flow cytometry analysis of DAPI-stained cells. The red dashed ellipses indicate the position of undifferentiated bacteria as in culture (not shown) or in the *dnf1* mutant nodules (lower-left ellipse) or fully mature bacteroids as in the A17 wild-type nodules (top-right ellipse). (M to X) The extent of methylation of (M to R) GANTC or (S to X) TCGA(N_8_)TCGA motifs across the *E. meliloti* FSM-MA chromosome, shown using a 10-kb sliding window. The red lines are polynomial regression lines calculated in R using the “rlm” method and the formula “y∼poly(x,2).” Data for pSymB and pSymA are shown in Fig. S20 and S21 at doi.org/10.6084/m9.figshare.16556205.

To further explore changes in CcrM activity during bacteroid differentiation, we took advantage of a collection of *M. truncatula* mutant plant lines (*dnf1*, *dnf2*, *dnf4*, *dnf5*, *dnf7*) whose nodules contain bacteria blocked at various stages of differentiation (see Fig. S19 at doi.org/10.6084/m9.figshare.16556205 for photographs of the plants) (51–57). Microscopy and flow cytometry data were consistent with past work (57) showing that bacteroids were blocked at the earliest to latest stages of differentiation in mutant plant lines in the order *dnf1* → *dnf5* → *dnf2* → *dnf7* → *dnf4* (Fig. 6). Nodule bacteria of *M. truncatula dnf1* mutant plants were small, with one or two haploid genome copies per cell (i.e., ploidy level = 1 or 2) (Fig. 6A and G), suggesting that the cell population was dominated by actively dividing cells that had not yet begun differentiation. Indeed, the GANTC methylation pattern of these cells (Fig. 6M and Fig. S20 and S21 at doi.org/10.6084/m9.figshare.16556205) resembled the frowning pattern of exponentially growing free-living cells (Fig. 5A). Although the nodule bacteria of *M. truncatula dnf5* mutant plants were also small and undifferentiated into bacteroids, the majority of cells had a ploidy level of 1 (Fig. 6B and H), suggesting these cells had ceased replication but had not yet begun the process of endoreduplication. GANTC methylation was consistently high across the chromosome of bacteria purified from *dnf5* nodules (Fig. 6N), similar to stationary-phase free-living cells (Fig. 6B) and indicating that terminal differentiation is preceded by full GANTC methylation.

The nodule bacteria purified from *M. truncatula dnf2* mutant nodules were a mix of undifferentiated and partially differentiated bacteroids, which were polyploid to an extent similar to bacteroids purified from wild-type A17 nodules (Fig. 6I compared to Fig. 6L); however, their cell size was much smaller (Fig. 6C compared to Fig. 6F). This was similar to differentiating bacteroids purified from *M. truncatula* and *M. sativa* zone II nodule sections, many of which had high ploidy without a corresponding increase in cell size (Fig. S11 and S13 at doi.org/10.6084/m9.figshare.16556205). The GANTC methylation pattern of bacteroids from *dnf2* nodules (Fig. 6O) was also similar to that of zone II nodule sections. There was a consistently high extent of GANTC methylation across the chromosome averaging 0.870, which was less than that of bacteroids purified from *dnf5* nodules (0.956) but higher than that of bacteroids purified from wild-type A17 nodules (0.804) (Table S5 at doi.org/10.6084/m9.figshare.16556205), and without the smiling pattern. The nodule bacteria purified from *M. truncatula dnf7* and *dnf4* nodules also contained a mix of undifferentiated bacteria and fully differentiated bacteroids (Fig. 6D, E, J, and K), with the number of undifferentiated bacteria greater in *dnf7* nodules compared to *dnf4* nodules. The GANTC methylation pattern of bacteroids purified from *dnf7* and *dnf4* nodules was similar to that of bacteroids purified from A17 nodules (Fig. 6P and Q). Overall, we interpret the data from bacteroids purified from section nodules and *M. truncatula dnf* mutant nodules as suggesting that CcrM is dysregulated during terminal bacteroid differentiation and that CcrM is constitutively active during endoreduplication.

### Chromosome, pSymB, and pSymA sequencing depth are unequal in *E. meliloti* bacteroids.

We noticed that in each bacteroid sample, the average extent of GANTC methylation for the chromosomes of the two strains was lower (by 0.04 to 0.13) than that of pSymA or pSymB, and unlike the chromosome, the extent of GANTC methylation was relatively constant across pSymA and pSymB (Fig. S14 to S17 compared to Fig. 4 and 5). These results suggest that, unlike in free-living cells, replication of the three replicons is not well coordinated during terminal differentiation. In agreement with this hypothesis, the mean sequencing depth across pSymA and pSymB was on average ∼33% lower than that of the chromosome in all replicates of the *E. meliloti* whole-nodule bacteroid samples (Table 2). Similarly, the mean sequencing depth across pSymA and pSymB was on average ∼23% lower than that of the chromosome for the polyploid bacterial cell populations purified from *M. truncatula dnf2*, *dnf7*, and *dnf4* mutant nodules, but not for the haploid/diploid bacterial cell populations purified from *M. truncatula dnf1* and *dnf5* mutant nodules (Table 2). Assuming sequencing depth is correlated with copy number, this observation suggests that *E. meliloti* bacteroids carry approximately two copies each of pSymA and pSymB per three copies of the chromosome.

**TABLE 2:** Relative sequencing depth of each *E. meliloti* replicon

Strain	Condition	Relative mean sequencing depth^*a*^		
		Chromosome	pSymA	pSymB
Rm2011	Mid-exponential phase	1.00 ± 0.00	1.00 ± 0.03	0.98 ± 0.03
Rm2011	Stationary phase	1.00 ± 0.00	1.06 ± 0.02	1.02 ± 0.03
Rm2011	*M. sativa* bacteroids	1.00 ± 0.00	0.64 ± 0.02	0.62 ± 0.02
FSM-MA	Mid-exponential phase	1.00 ± 0.00	1.02 ± 0.03	0.87 ± 0.05
FSM-MA	Stationary phase	1.00 ± 0.00	1.10 ± 0.00	1.01 ± 0.03
FSM-MA	*M. sativa* bacteroids	1.00 ± 0.00	0.79 ± 0.04	0.71 ± 0.02
FSM-MA	*M. truncatula* bacteroids	1.00 ± 0.00	0.66 ± 0.02	0.59 ± 0.01
FSM-MA	*dnf1* bacterial cells	1.00	0.98	0.90
FSM-MA	*dnf5* bacterial dells	1.00	0.96	0.94
FSM-MA	*dnf2* bacterial dells	1.00	0.81	0.73
FSM-MA	*dnf7* bacterial dells	1.00	0.82	0.71
FSM-MA	*dnf4* bacterial dells	1.00	0.80	0.73
FSM-MA	A17 bacteroids	1.00	0.76	0.67

a Sequencing depth is presented relative to the sequencing depth of the chromosome in the same sample. Values are the means of triplicate samples ± standard deviation, except for the lower half of the table, for which numbers are based on a single replicate.

## DISCUSSION

In this study, we examined the genome-wide DNA methylation patterns in the free-living cells of four *Ensifer* strains, and in bacteroids of two *E. meliloti* strains, and detected a total of six methylated motifs. We were able to predict cognate MTases for most of the motifs based on genome annotations, the exception being the WNCCGATG motif of *E. adhaerens* OV14. The CGCA(N_5_)GTG motif of *E. meliloti* Rm2011 is presumably methylated by Smc02296 (HsdM), a predicted m6A MTase belonging to the HsdRSM type I R-M system that is known to be functional and reduce transformation efficiency (58, 59). The RCGCCTC motif of *E. meliloti* Rm2011 is possibly methylated by Smc03763, a predicted cytosine MTase located upstream of the gene *vsr*, which putatively encodes a very short patch repair protein. However, as MTases encoded upstream of *vsr*-like genes generally create m5C modifications—not m4C modification as in the RCGCCTC motif—it is possible that methylation of RCGCCTC may instead occur by an unidentified MTase (60). Neither of these proteins are found in the other three strains examined here. The motifs TCGA(N_8_)TCGA of *E. meliloti* FSM-MA and CAGA(N_7_)GTTG of *E. fredii* NGR234 are likely methylated by SMB554_16155 and NGR_c01340, respectively, which are 88% identical at the amino acid level and appear to function as part of R-M systems. Homologs of these two proteins are not found in the other two strains.

Except for GANTC, each methylated motif was detected as methylated only in a single strain. Moreover, MTases, apart from CcrM, are not conserved among *E. meliloti* strains. The lack of conservation suggests that most DNA MTases do not have a major regulatory role in the genus *Ensifer*, aside from CcrM and its role in cell cycle regulation. Supporting this conclusion, no motif was enriched in the promoter regions of symbiosis, carbon source, or cell cycle-regulated genes, and we did not detect any motifs that were methylated specifically in bacteroids. However, we cannot rule out that one or more methylated motifs may influence specific gene expression during free-living growth, differentiation, or N_2_-fixation through extended motifs or proximity to other promoter elements, similar to the interplay between CcrM and GcrA during cell cycle regulation in C. crescentus (20, 46).

As previously published studies have provided evidence for a cell cycle transition occurring during terminal bacteroid differentiation (26), we were particularly interested in CcrM, a cell cycle-regulated MTase that is broadly conserved in the *Alphaproteobacteria*, and its cognate DNA motif, GANTC. By identifying GANTC sites in the promoter regions of a previously determined set of 462 cell cycle-regulated genes (45), we defined a candidate CcrM regulon in *E. meliloti* consisting of 111 genes. However, further studies are required to better delineate the CcrM regulon in *E. meliloti*, as individual GANTC sites are not diagnostic of a specific gene being regulated by CcrM; GANTC sites were found in the promoter regions of 904 transcripts that did not display cell cycle regulation, and the promoter regions of cell cycle-regulated genes were not enriched in GANTC sites relative to the whole *E. meliloti* genome. Studies in C. crescentus suggest that the impact of the fully or hemi-methylated status of GANTC sites on gene expression is mediated, at least in part, through modulating the activity of the transcriptional regulator GcrA (20, 61). However, not all promoter sites containing a GANTC motif are regulated by GcrA in C. crescentus, with the relationship dependent on an extended YGAKTCK motif and the precise position of this motif relative to other promoter elements (61). Likely, CcrM-mediated gene regulation in the genus *Ensifer* is also dependent upon additional sequence elements beyond the GANTC motif. Nevertheless, as a recent study in the alphaproteobacterium Brevundimonas subvibrioides suggested (46), the CcrM and GcrA regulons as well as the GcrA-binding motif can show very little overlap between species, and therefore they will have to be defined experimentally in *E. meliloti*.

Consistent with past observations (18), GANTC sites were underrepresented in the genomes of 157 *Ensifer* strains, particularly within coding regions. More surprising, however, was the strong difference in the frequency of GANTC sites between the previously defined (47) symbiotic and nonsymbiotic clades in the genus *Ensifer*, with the frequency of GANTC sites being ∼60% higher in the symbiotic clade. Although further work is required to understand the biological significance of the greater frequency of GANTC sites in the symbiotic clade, it is tempting to speculate that it is associated with legume symbiosis.

Our data are consistent with CcrM activity differing during terminal bacteroid differentiation compared to free-living cells. The overall moderate to high rates of GANTC methylation in all *E. meliloti* bacteroid samples, coupled with the lack of a chromosome-wide pattern in the *E. meliloti* FSM-MA zone II sample, leads us to hypothesize that CcrM remains constitutively active throughout most of terminal differentiation. This hypothesis is supported by the results for nodule bacteria purified from *M. truncatula dnf* mutant nodules, which showed that differentiation is preceded by full GANTC methylation and that GANTC methylation remains high (but moderately lower) during endoreduplication, followed by another moderate drop in GANTC methylation in late stages of differentiation. Considering that overexpression of CcrM can give rise to bacteroid-like morphology in free-living cells (14), we hypothesize that constitutive CcrM MTase activity is one (of potentially multiple) factor(s) driving polyploidization of bacteroids (Fig. 2). However, further studies monitoring CcrM abundance and artificially manipulating *ccrM* expression throughout bacteroid differentiation are required to conclusively determine if CcrM is constitutively active during terminal differentiation and the importance of this activity to the promotion of endoreduplication.

CcrM activity is confined to a short window in the cell cycle since the *ccrM* gene is expressed in the late phase of genome replication (45) and, at least in C. crescentus, the CcrM protein is degraded by the Lon protease prior to cell division (62). Thus, constitutive CcrM MTase activity in differentiating bacteroids could be obtained through an aberrant expression of the gene or, alternatively, a lack of proteolytic degradation of the CcrM protein. In agreement with the latter possibility, Lon protease was identified as a target of the NCR247 peptide (34). NCR247 is one of the ∼600 NCR peptides produced by *M. truncatula* that induce bacteroid terminal differentiation, and studies suggest that NCR247 does so at least in part by perturbing expression of genes involved in cell cycle regulation (33). It is tempting to speculate that NCR peptides such as NCR247 inhibit Lon protease activity posttranslationally, thereby stabilizing CcrM and triggering bacteroid differentiation. However, the CcrM MTase does not appear to remain active in fully differentiated bacteroids, with the lower GANTC methylation near the chromosomal *ter* regions suggesting that loss of CcrM MTase activity occurs slightly prior to completion of genome endoreduplication (model provided as Fig. 2). These hypotheses are consistent with *M. truncatula*-*E. meliloti* nodule zone-specific RNA-sequencing data (24), which showed that *ccrM* expression in the root distal portion of zone II is ∼2-fold higher than in the root proximal portion of zone II and ∼10-fold higher than in zone III. The ∼10-fold difference in *ccrM* expression across nodule zones suggests to us that the level of *ccrM* expression during early stages of bacteroid differentiation is biologically significant, a prerequisite for the constitutive CcrM activity that we hypothesize.

Our analyses also provide insight into the genome replication dynamics of *E. meliloti* during free-living growth and terminal bacteroid differentiation. Notably, flow cytometry data of *E. meliloti* bacteroids purified from zone II nodule sections and *M. truncatula dnf2* nodules suggest that endoreduplication and cell enlargement largely occur subsequently, not concurrently. Genome replication might be a much faster process than cell growth or, alternatively, endoreduplication might be required to drive cell enlargement. Moreover, our data are consistent with a loss of coordination of replication of the three replicons during terminal bacteroid differentiation, leading to unequal copy numbers, with two copies of pSymA and pSymB per three copies of the chromosome in bacteroids. This differs from free-living cells, where the copy number of the three replicons was approximately equal based on average sequencing depth. We hypothesize that this is a consequence of pSymA and pSymB encoding their own replication proteins and having independent regulation of replication initiation and copy number from that of the chromosome (63, 64). The relative change in replicon copy number occurs concomitantly with differentiation and polyploidization, as supported by the relative abundance of the replicons differing in nodule bacterial cells that have experienced endoreduplication (i.e., cells retrieved from *M. truncatula dnf2*, *dnf7*, and *dnf4* mutant nodules) but not in bacterial cells that had not yet undergone endoreduplication (i.e., cells retrieved from *M. truncatula dnf1* and *dnf5* mutant nodules). In contrast to our results, a previous comparison of the relative abundance of the three replicons in free-living *E. meliloti* Rm1021 versus bacteroids did not detect differences using comparative genome hybridization with microarrays (26). We believe that the difference between our present data and the previous analysis is due to the subtlety of the differences and the lower sensitivity of the microarray hybridization method compared to high-throughput sequencing.

We also observed that during free-living exponential growth, the extent of GANTC methylation at the *ori* of pSymA and pSymB is higher than at the *ori* of the chromosome, while the *ter* of the pSymA and pSymB has a slightly lower extent of GANTC methylation than the *ter* of the chromosome. As GANTC methylation occurs at a fixed stage of the cell cycle corresponding to the end of chromosome replication, our observations indicate that pSymA and pSymB replication is initiated later in the cell cycle than initiation of chromosome replication, while their replication terminates slightly before completion of chromosomal replication and the activation of CcrM (model provided as Fig. 2). These results provide additional support for the notion of spatiotemporal regulation of DNA replication and partitioning in the multipartite *E. meliloti* genome as proposed previously (45, 65). Similarly, replication of chromosome II of Vibrio cholerae is delayed relative to chromosome I, leading to the replication of these two replicons terminating at approximately the same time (66). Thus, coordinating the timing or replication termination may be a general feature of multipartite genomes.

## MATERIALS AND METHODS

### Experimental design.

The overall experimental design is summarized in Fig. S1 at doi.org/10.6084/m9.figshare.16556205. Genomic DNA was isolated from four wild-type *Ensifer* strains to explore how DNA methylation varies across this genus; to allow direct comparison, the four strains were grown to mid-exponential phase in minimal medium with succinate as a carbon source. To investigate how DNA methylation patterns differ between actively dividing and nondividing cells, genomic DNA was isolated from *E. meliloti* Rm2011 grown to either mid-exponential phase or stationary phase. Genomic DNA was isolated from *E. meliloti* Rm2011 grown to mid-exponential phase with either a glycolytic (sucrose) or gluconeogenic (succinate) carbon source to investigate whether DNA methylation may play a role in regulating carbon metabolism. Furthermore, an *E. meliloti* Rm2011 derivative lacking the pSymA and pSymB replicons (named RmP3496) was studied to gain insight into whether these replicons contribute to DNA methylation patterns in *E. meliloti* Rm2011; this strain was grown with sucrose (instead of succinate), as RmP3496 lacks the succinate transporter.

In addition to the free-living samples, *E. meliloti* bacteroid samples purified from legume nodules were collected to investigate changes in DNA methylation during bacteroid differentiation and nitrogen fixation. To do so, *E. meliloti* Rm2011 and *E. meliloti* FSM-MA bacteroids were isolated from *M. sativa* whole nodules. *E. meliloti* FSM-MA bacteroids were additionally purified from *M. truncatula* whole nodules to examine the impact of the host plant on bacteroid DNA methylation patterns. *E. meliloti* Rm2011 bacteroids were only isolated from *M. sativa* nodules, as unlike FSM-MA, Rm2011 forms a poor symbiosis with *M. truncatula* (48, 49). Moreover, *E. meliloti* Rm2011 and *E. meliloti* FSM-MA bacteroids were isolated from *M. sativa* nodule sections (sectioned at the white-pink border to separate the root distal infection and differentiation zone II [white] from the root proximal nitrogen-fixing zone III [pink]) to facilitate an analysis of how DNA methylation patterns differ between differentiating bacteroids and fully differentiated and nitrogen-fixing bacteroids. This was followed by isolation of *E. meliloti* FSM-MA bacteroids from whole nodules of *M. truncatula* mutant lines (*dnf1*, *dnf2*, *dnf4*, *dnf5*, *dnf7*) to investigate DNA methylation patterns in nodule bacteria blocked as various stages of differentiation.

### Bacterial strains and growth conditions.

The bacterial strains used in this work are listed in Table S6 at doi.org/10.6084/m9.figshare.16556205. All strains were routinely grown on tryptone-yeast (TY) with 2 μM CoCl_2_, as it was required for *E. meliloti* RmP3496 (42). The MM9 minimal medium (42) consisted of the following: 40 mM MOPS (morpholinepropanesulfonic acid), 20 mM KOH, 19.2 mM NH_4_Cl, 85.6 mM NaCl, 2 mM KH_2_PO_4_, 1 mM MgSO_4_, 0.25 mM CaCl_2_, 1 μg mL^−1^ biotin, 42 nM CoCl_2_, 38 μM FeCl_3_, 10 μM thiamine-HCl, and either 10 mM sucrose (MM9-sucrose) or 20 mM disodium succinate (MM9-succinate). Prior to inoculation of plants with *E. meliloti*, the strains were grown in yeast extract-beef broth (YEB) medium (67).

### DNA isolation from free-living cells.

Overnight cultures of all strains grown in MM9-succinate or MM9-sucrose medium were diluted into 10 mL of the same medium to a starting optical density at 600 nm (OD_600nm_) of 0.025 (0.05 for RmP3496) and incubated overnight at 30°C with shaking (130 rpm). The next day, cultures were diluted into 40 mL of the same medium in 100-mL flasks to the OD_600nm_ values listed in Table S7 at doi.org/10.6084/m9.figshare.16556205. To obtain mid-exponential-phase samples, cultures were harvested after 15.5 to 16 h of growth at OD_600nm_ values between 0.37 and 0.69 (Table S7 at doi.org/10.6084/m9.figshare.16556205). To obtain stationary-phase samples, cultures were harvested after 24 h of growth at OD_600nm_ values of ∼1.4. In all cases, cultures were streaked on TY plates to check for contamination and then centrifuged (8,200 × *g*, 10 min, 4°C); the full 40 mL was centrifuged for mid-exponential-phase cultures, whereas only 15 mL was centrifuged for stationary-phase cultures. Most of the supernatant was discarded, and the pellet was resuspended in the remaining volume, transferred to a 2-mL tube and centrifuged again (16,200 × *g*, room temperature, 1 min), and the supernatant was discarded. Three biological replicates, each starting from a separate overnight culture, were performed. DNA was isolated using phenol:chloroform extractions and ammonium acetate precipitations as described elsewhere (68), and the DNA pellets (following RNase A treatment) were resuspended in 200 μL of 10 mM Tris-HCl, pH 8.5.

### DNA isolation from bacteroids.

*M. sativa* cv. Gabès and *M. truncatula* cv. A17 were the wild-type plants used for all experiments. *M. truncatula dnf1*, *dnf2*, *dnf4*, *dnf5*, and *dnf7* mutants (51), derived from the A17 wild type, were used for collection of bacteroids blocked at various stages of differentiation. Seeds were scarified, surface sterilized, and germinated on Kalys agar as described previously (49). First, 50 mL of overnight cultures of *E. meliloti* Rm2011 or FSM-MA, grown in YEB, were centrifuged (4,000 × *g*, 20 min, room temperature) and resuspended in ∼1,200 mL of sterile, distilled water to obtain a cell suspension at an OD_600nm_ of ∼0.1. Germinated seedlings were immersed for 1 h in the appropriate rhizobial cell suspension and then planted in a perlite:sand (2:1) mixture. Plants were grown in a greenhouse for five to six weeks, with occasional watering with a 1 g L^−1^ nutrient solution (PlantProd solution [N-P-K, 0-15-40]).

For whole-nodule samples of wild-type plants, pink nodules were collected from 53 to 60 plants per replicate 34- to 35-day postinoculation; in the case of *dnf* mutants (and a matched wild-type A17 sample), nodules were collected from ∼105 plants per genotype 23 to 24 days postinoculation. Nodules were collected from the roots and stored in tubes in liquid nitrogen until collection was complete, at which point they were stored at −80°C until use. For sectioned nodule samples, pink nodules were collected from 103 *M. sativa* plants for each of the microsymbionts 35 to 40 days postinoculation. Nodules were manually sectioned at the white-pink border. Nodule sections were stored in tubes over dry ice or liquid nitrogen until collection was complete, at which point they were stored at −80°C until use. Average plant shoot dry weights for all samples are listed in Table S8 at doi.org/10.6084/m9.figshare.16556205. Bacteroids were isolated from the nodule samples using Percoll gradient centrifugation as described elsewhere (26). The recovered bacteroids were resuspended in 50 to 100 μL of bacteroid extraction buffer (BEB; 125 mM KCl, 50 mM Na-succinate, 50 mM TES [*N*-tris(hydroxymethyl)methyl-2-aminoethanesulfonic acid], pH 7.0) and either used immediately for microscopy, flow cytometry, and DNA isolation or stored at −80°C until use.

Nucleic acids were initially purified from most bacteroid samples using the Epicentre MasterPure complete DNA and RNA purification kit, following the protocol for DNA isolation from cell samples; the exceptions were bacteroids purified from *dnf* mutant nodules (and the matched wild-type A17 sample), for which nucleic acids were isolated by using phenol:chloroform extractions followed by ammonium acetate DNA precipitations as described elsewhere (68). For sectioned-nodule samples, pure DNA was isolated by using the manufacturer’s protocol for the complete removal of RNA. For whole-nodule samples, the isolated DNA was further purified by treating the nucleic acid samples with RNase A, after which pure DNA was isolated by using phenol:chloroform extractions followed by ammonium acetate DNA precipitations or, alternatively, using the MasterPure DNA cleanup protocol for the DNA from *dnf* mutant nodules and the matched wild-type A17 sample. In all cases, the final DNA pellets were resuspended in 200 μL of 10 mM Tris-HCl, pH 8.5. Three biological replicates were performed for bacteroids isolated from most whole nodules, whereas only one replicate was performed for bacteroids isolated from sectioned nodules or *dnf* mutant nodules (and the matched wild-type A17 sample) due to low quantities of starting materials.

### DNA sequencing, modification detection, and motif analysis.

DNA sequencing was performed at the U.S. Department of Energy Joint Genome Institute (JGI) or in-house at the University of Florence (the stationary-phase samples and *dnf* mutant nodules and the matched wild-type A17 sample) using Pacific Biosciences (PacBio) sequencing technology (69). Genomic DNA was sheared to 3 kb using an LS220 (Covaris, Inc., Woburn, MA, USA) or 15 kb (for stationary-phase samples and bacteroids isolated from *dnf* mutant nodules and the matched wild-type A17 sample) using g-TUBEs (Covaris, Inc.). Sheared DNA was treated with exonuclease to remove single-stranded ends and DNA damage repair mix followed by end repair and ligation of barcoded blunt adapters using the SMRTbell template prep kit 2.0 (PacBio, Menlo Park, CA, USA). Libraries were purified with AMPure PB beads (Beckman Coulter, Brea, CA, USA), and three or eight libraries with different barcodes were pooled at equimolar ratios and purified with AMPure PB beads. For most samples, SMRTbell template libraries were prepared using a Sequel binding kit 3.0 (PacBio, Menlo Park, CA, USA) and sequenced on a Sequel instrument using a v3 or v4 sequencing primer, 1 M v3 SMRT cells, and version 3.0 sequencing chemistry with 1 × 360 or 1 × 600 sequencing movie run times. The exceptions were the *E. meliloti* Rm2011 zone II and *E. meliloti* FSM-MA zone III bacteroid samples. For these samples, SMRTbell template libraries were prepared using a Sequel II binding kit 2.0 (PacBio) and then sequenced on a Sequel II instrument using the tbd-sample-dependent sequencing primer, 8 M v1 SMRT cells, and version 2.0 sequencing chemistry with 1 × 900 sequencing movie run times.

DNA modification detection and motif analysis were performed using the SMRT Link software (PacBio, Menlo Park, CA, USA). Briefly, raw reads were filtered using SFilter to remove short reads and reads derived from sequencing adapters. Filtered reads were aligned against the appropriate reference genome (Table S2 at doi.org/10.6084/m9.figshare.16556205) using BLASR (70), and modified sites were then identified through kinetic analysis of the aligned DNA sequence data (71); the number of mapped bases per sample is provided in Table S2 at doi.org/10.6084/m9.figshare.16556205. Modified sites were then grouped into motifs using MotifFinder. These motifs represent the recognition sequences of MTase genes active in the genome (72). Downstream analyses were performed using custom Perl and R scripts.

### Flow cytometry.

Flow cytometry was performed as described previously (26). Freshly prepared bacteroid samples were diluted in 200 μL of BEB, heat-treated for 10 min in a 70°C water bath, and then stained with the DNA-binding dye diamidino-2-phenylindole (DAPI). Cell size and ploidy level of the bacteroid samples were determined using flow cytometry with a Beckman Coulter CytoFLEX S instrument. Measurements consisted of 50,000 cells. Data analysis was performed using the CytExpert 2.2.0.97 software.

### Fluorescence microscopy.

One μL of each freshly prepared bacteroid sample was mixed with 1 μL of 50 μg mL^−1^ DAPI or with both 1 μL of 50 μg mL^−1^ DAPI and 1 μL of 100 μg mL^−1^ propidium iodide (PI), which are both DNA binding dyes. Samples were visualized at ×100 magnification under oil immersion using a Nikon Eclipse 80*i* fluorescence microscope with the NIS-Elements BR 4.00.01 software and a Digital Sight DS-U3 camera.

### Phylogenetic analysis.

The nucleotide fasta files of representative *Ensifer* species were downloaded from the National Centre for Biotechnology Information (NCBI) genome database. A core gene phylogeny was constructed using a previously prepared pipeline (73) reliant on the use of Roary 3.11.3 (74), Prokka 1.12-beta (75), PRANK 140110 (76), trimAl (77), and RAxML 8.2.9 (78). The phylogeny was visualized with the iTOL webserver (79). Identification of nodulation (*nodABC*) and nitrogenase genes (*nifHDK*) was performed with a published pipeline (73) reliant on the use of HMMER 3.1b2 (80), and the Pfam-A 31.0 (81) and TIGRFAM 15.0 (82) databases.

### Data availability.

Most sequencing data are available through the JGI Genome Portal (genome.jgi.doe.gov/portal/) under proposal 503835, as well as through the NCBI (for BioSample accession numbers, see Table S2 at doi.org/10.6084/m9.figshare.16556205). The data for stationary-phase cultures and bacteroids isolated from *dnf* mutant nodules are available only through the NCBI (BioProject accession no. PRJNA706182 and PRJNA705832; for BioSample accession numbers, see Table S2 at doi.org/10.6084/m9.figshare.16556205). Raw flow cytometry FCS files are available as Data set S3 at doi.org/10.6084/m9.figshare.16556205. All custom scripts to perform the analyses described in this study are available through GitHub (github.com/diCenzo-Lab/003_2021_Ensifer_DNA_methylation).
